# Improving the robustness and stability of a machine learning model for breast cancer prognosis through the use of multi-modal classifiers

**DOI:** 10.1038/s41598-023-30143-8

**Published:** 2023-03-11

**Authors:** Nikhilanand Arya, Sriparna Saha, Archana Mathur, Snehanshu Saha

**Affiliations:** 1 Department of Computer Science & Engineering, Indian Institute of Technology, Patna, Bihar 801106 India; 2 Department of Information Science & Engineering, Nitte Meenkashi Institute of Technology, Bangalore, 560064 India; 3 APPCAIR & CSIS, Birla Institute of Technology and Science, Pilani-Goa Campus, Pilani, Goa 403726 India

**Keywords:** Computational biology and bioinformatics, Machine learning, Breast cancer

## Abstract

Breast cancer is a deadly disease with a high mortality rate among PAN cancers. The advancements in biomedical information retrieval techniques have been beneficial in developing early prognosis and diagnosis systems for cancer patients. These systems provide the oncologist with plenty of information from several modalities to make the correct and feasible treatment plan for breast cancer patients and protect them from unnecessary therapies and their toxic side effects. The cancer patient’s related information can be collected using various modalities like clinical, copy number variation, DNA-methylation, microRNA sequencing, gene expression, and histopathological whole slide images. High dimensionality and heterogeneity in these modalities demand the development of some intelligent systems to understand related features to the prognosis and diagnosis of diseases and make correct predictions. In this work, we have studied some end-to-end systems having two main components : (a) dimensionality reduction techniques applied to original features from different modalities and (b) classification techniques applied to the fusion of reduced feature vectors from different modalities for automatic predictions of breast cancer patients into two categories: short-time and long-time survivors. Principal component analysis (PCA) and variational auto-encoders (VAEs) are used as the dimensionality reduction techniques, followed by support vector machines (SVM) or random forest as the machine learning classifiers. The study utilizes raw, PCA, and VAE extracted features of the TCGA-BRCA dataset from six different modalities as input to the machine learning classifiers. We conclude this study by suggesting that adding more modalities to the classifiers provides complementary information to the classifier and increases the stability and robustness of the classifiers. In this study, the multimodal classifiers have not been validated on primary data prospectively.

## Introduction

The breast is an essential glandular organ in the female body. It comprises fat, connective, and breast tissues containing lobes, lobules, and milk glands. Rapid and uncontrolled growth of these tissues, more specifically breast cells, causes a prevalent and fatal disease, breast cancer. It is not worrisome if the growth is non-invasive and confined within the milk ducts and lobules, but it becomes deadly in invasive cases when it spreads to adjacent and non-adjacent tissues or organs. The invasive one is also identified as metastatic breast cancer. According to the statistics provided by Global Cancer Observatory (GCO), breast cancer has a significant contribution (11.7%) in the estimated cancer cases (19,292,789) globally for the year 2020. These statistics have considered patients of all sexes and ages but stopping at only female cancerous patients, the contribution of breast cancer cases increases to 24.5%. The deadly nature of this disease is observed in an estimated 1 million breast cancer deaths out of 3.2 million breast cancer cases by the year 2040. We need a better prognosis and diagnosis system that integrates qualitative and quantitative information from histology and genomics, respectively, to predict clinical outcomes in light of the grim future of breast cancer. By avoiding the harmful side effects of various cancer-related therapies, preventing overtreatment, and thereby lowering economic costs^1^ , more effectively including and excluding patients in a randomized trial, and developing palliative care and hospice care systems^2^, a precise and timely prognosis of cancer patients can help both patients and oncologists in choosing the appropriate treatment. Hence, it is essential to know the severity of the disease and the life expectancy of a breast cancer patient at an early stage of treatment. It does not seem easy to predict the patient’s survival due to the heterogeneous and complex nature of cancer-related data coming from genetic and histopathological sources. The clinical outcomes also vary significantly from patient to patient, which makes it a further challenging task^3^. The standard survival cut-off for any disease is five years because it takes this much time to label the patient’s survivability. It can be validated from earlier research where authors have utilized a 5-year criterion to determine the cohort’s survivability^4–7^. Although survival rates have increased dramatically in recent years due to breast cancer’s complexity, individual differences in the 5-year survival rate remain. Short-term and long-term survivors are defined as patients with life expectancies that fall below and over the survival cut-off. The availability of large-scale multi-modal data such as METABRIC^8^, The Cancer Genome Atlas (TCGA)^9^, derived from advanced histopathology and next-generation genome sequencing is supportive in the development of automated and artificially intelligent machine learning architectures. These architectures can be developed for different tasks of breast cancer diagnosis and prognosis and help oncologists with precise treatment plans. As medical research has matured, a variety of cutting-edge and distinctive methods for leveraging big data analytic techniques in the development of survival prediction models have been created. Machine learning (ML) is capable of handling dependencies and nonlinear interactions between variables, requires no implicit assumptions and can learn data models on its own^10^. It excels at handling the vast majority of complex higher-order interactions that can be discovered in medical data. As a result, machine learning techniques have enormous potential for usage as cutting-edge health informatics tools in routine medical practices.

### Related work and motivations

In this section, we will look at past research on breast cancer prognosis and diagnosis, followed by the limitations that became motivations for our proposed work. The journey of uni-modal to multi-modal architectures in breast cancer prognosis and diagnosis starts from the usefulness of gene expression profiles derived with the help of microarray technology. The initial days of the research were based on identifying gene signatures or gene markers responsible for the disease. In the task of breast cancer prognosis, Van’t Veer et al.^11^ identified 70-gene markers with microarray data of very few primary breast cancer patients. Further, Van et al.^12^ validated the usefulness of these gene markers using a larger group of breast cancer patients. This uni-modal supervised classification task is done with the help of some popular machine learning techniques such as Support Vector Machine (SVM)^13^ and Random Forest (RF)^14^. Here, Xu et al.^13^ trained the SVM-based feature selection model using microarray data to identify the smaller but informative subset of signature genes responsible for breast cancer prognosis prediction. Shifting the research from traditional microarray profiles to histopathological tissue images, Nguyen et al.^14^ outperformed the previous results with the help of tissue images-based RF classifier, which has the power of multiple decision trees. This study combines Bayesian probability and RF for feature ranking and classification. Further, the exploration to identify the significance of more than one modality in the breast cancer prognosis started, and the integration of microarray profiles with either clinical profiles or histopathological data has found its way as bi-modal study. In terms of gene expression and clinical details integration, I-RELIEF^15^ was proposed as a feature selection algorithm to select hybrid signatures with three genes and two clinical markers for breast cancer prognosis; Gevaert et al.^16^ employed the bayesian network for the prognosis of lymph-node negative breast cancer over the pathological and gene expression data; Khademi et al.^17^ developed the probabilistic graphical model (PGM) and integrated two models exclusive of each other, which are based on microarray and clinical profiles. Not limiting the bi-modal study to gene expression and clinical profiles, ENCAPP^18^ (an elastic-net-based technique) used the protein-protein interaction network in collaboration with gene expression for getting the odds of survival in human breast cancer patients. The paradigm shift from uni to multi-modality can be validated in some of the recent works on breast cancer prognosis, and diagnosis^5–7^. Sun et al. also created the GPMKL^19^ approach based on the ensemble of multiple kernel functions, which integrates genomic data and pathological images to predict breast cancer prognosis.

Understanding aberrant images and tumor morphology has never been simpler because of advances in medical imaging technologies^20,21^. Assuming that pathological images may provide extra information about tumor features, the current study created some computational algorithms for forecasting cancer clinical outcomes based on those images. Wang et al.^22^ established an integrated framework for non-small cell lung cancer computer-aided diagnosis and survival analysis using suggestive markers from histopathology slides. Using pathological picture characteristics and gene expression profiles, Zhu et al.^23^ developed a prediction model for lung cancer survival. Using 2186 Hematoxylin and Eosin pathological whole-slide images (WSIs) of non-small cell lung cancer, Yu et al.^24^ retrieved 9879 significant picture attributes and utilized traditional classification methods to distinguish between short-term and long-term survivors. With the availability of histopathological images and the need for the research of survival analysis in the medical field in mind, Tang et al. created the CapSurv Capsule Network^25^. The foundation of this approach is a special function termed survival loss, specifically developed for the analysis of cancer patients’ survival rates.

A thorough assessment of the literature revealed that gene expression profiles are useful for predicting breast cancer prognosis. However, high-dimensional microarray data and gene correlation pose severe problems in identifying gene markers and predicting their prognosis. Any machine learning architecture suffers from the curse of dimensionality when combining multiple high-dimensional modality combinations. Due to the complexity and heterogeneity of this severe disease, there is currently not enough research on multi-modal breast cancer clinical outcome analysis, despite the encouraging results of the approaches discussed previously for other cancer prognoses. While this is going on, the ever-increasing number of variables from diverse data sources and the use of heterogeneous traits may make it very challenging to combine them effectively for predicting breast cancer survival. Despite several multi-modal studies for breast cancer, no study includes all six modalities (clinical, copy number variation, DNA-methylation, micro-RNA Sequencing, gene expression, and histopathological whole slide images) for survival classification. The current study investigates the impact of multiple modalities in machine learning-based breast cancer survival classifiers. But the raw features from all these modalities are very high-dimensional, consisting of redundant and noisy attributes. The high-dimensional feature space hampers the learning of the machine learning classifiers. Hence, we need to reduce the feature space by feature selection or extraction techniques. The reduced feature space boosts the learning of the model in terms of its generalization ability and computational complexity. So, the first task of our model is to extract the pertinent features from all the modalities using VAEs or PCAs, and the second task is to fuse the extracted features for the final classification.

In this work, we propose the following:

1. Investigation of six different modalities (clinical, copy number variation, DNA-Methylation, miRSeq, mRNASeq and Histopathological whole slide images) in the task of breast cancer survival prediction.
2. Log-cosh VAEs as dimensionality reduction technique for all the modalities, where L$_{1}$ or L$_{2}$ loss of VAE is replaced with *logcos*(*h*) loss function.
3. Six different PCAs as dimensionality reduction techniques for all the modalities.
4. Raw features, PCA projected features or Log-cosh VAEs extracted features as input to various machine learning classifiers (SVMs, Random Forest) for the survival estimation task.

## Dataset and methods

### Dataset

The study is focused on breast cancer prognosis prediction based on multiple modalities, and the most suitable dataset for this study is The Cancer Genome Atlas Program for Breast Cancer (TCGA-BRCA). We have downloaded this data from https://xenabrowser.net/datapages/. TCGA-BRCA dataset has total of six modalities for each breast cancer patient. It includes clinical (${{\textbf {m}}}_{{cln}}$) details of 1236 patients, copy number variations (${{\textbf {m}}}_{{cnv}}$) of 1079 patients, DNA-Methylation (${{\textbf {m}}}_{{dna}}$) with common attributes between Illumina Human Methylation 450 and Illumina Human Methylation 27 of 1227 patients, miRSeq (${{\textbf {m}}}_{{mir}}$) with IlluminaGA and IlluminaHiseq of 1188 patients, mRNASeq (${{\textbf {m}}}_{{mrna}}$) of 1215 patients, and Histopathological Whole Slide Images (${{\textbf {m}}}_{{wsi}}$) of 1068 patients. The raw dataset does not have all the patients present throughout various modalities resulting in incomplete-multi-view data. In a multi-modal learning framework, learning of any machine learning model becomes troublesome due to incomplete multi-view scenarios^26^. Hence, we have selected the complete multi-view data among all the possible combinations of modalities using the intersection technique based on common patient ids. The final selected patients for all possible combinations are presented in Table 1.

**Table 1:** Number of common samples for various combinations of modalities

# samples	Modalities
	**Quad-modal: **(cln_cnv_mir_wsi, cln_cnv_mir_wsi,cnv_dna_mir_wsi, cnv_mir_mrna_wsi)
1035	**Penta-modal:** (cln_cnv_dna_mir_wsi, cln_cnv_mir_mrna_wsi, cnv_dna_mir_mrna_wsi)
	**Hexa-modal:** (cln_cnv_dna_mir_mrna_wsi)
1048	**Quad-modal: ** (cln_dna_mir_wsi, cln_dna_mir_wsi, dna_mir_mrna_wsi)
	**Penta-modal:** (cln_dna_mir_mrna_wsi)
1049	**Bi-modal:** (mir_wsi) **Tri-modal:** (cln_mir_wsi, mir_mrna_wsi) **Quad-modal:** (cln_mir_mrna_wsi)
	**Tri-modal:** (cln_cnv_wsi, cnv_dna_wsi, cnv_mrna_wsi)
1053	**Quad-modal:** (cln_cnv_dna_wsi, cln_cnv_mrna_wsi, cnv_dna_mrna_wsi) **Penta-modal:** (cln_cnv_dna_mrna_wsi)
1058	**Tri-modal:**(cnv_mir_mrna) **Quad-modal:** (cln_cnv_mir_mrna, cnv_dna_mir_mrna) **Penta-modal:** (cln_cnv_dna_mir_mrna)
1060	**Bi-modal:**(cnv_mir), **Tri-modal:** (cln_cnv_mir, cnv_dna_mir), **Quad-modal:**(cln_cnv_dna_mir)
1067	**Tri-modal:**(cln_dna_wsi, dna_mrna_wsi) **Quad-modal:** (cln_dna_mrna_wsi)
1068	**Uni-modal:**(wsi), **Bi-modal:** (cln_wsi, mrna_wsi), **Tri-modal:** (cln_mrna_wsi)
1077	**Bi-modal:**(cnv_mrna)**Tri-modal:** (cln_cnv_mrna, cnv_dna_mrna) **Quad-modal:** (cln_cnv_dna_mrna)
1079	**Uni-modal:** (cnv), **Bi-modal:** (cln_cnv, cnv_dna) **Tri-modal:**(cln_cnv_dna)
1177	**Tri-modal:** (dna_mir_mrna), **Quad-modal:** (cln_dna_mir_mrna)
1179	**Bi-modal:**(dna_mir), **Tri-modal:** (cln_dna_mir)
1186	**Bi-modal:** (mir_mrna), **Tri-modal:** (cln_mir_mrna)
1188	**Uni-modal:** (mir), **Bi-modal:** (cln_mir)
1206	**Bi-modal:** (dna_mrna), **Tri-modal:**(cln_dna_mrna)
1215	**Uni-modal:**(mrna), **Bi-modal:** (cln_mrna)
1227	**Uni-modal:** (dna), **Bi-modal:**(cln_dna)
1236	**Uni-modal:** (cln)

For ${{\textbf {m}}}_{{cln}}$ modality, we select a total of 21 categorical features consisting of demographic details (age, gender, race, ethnicity) and primary cancer diagnostic details (history of radiation therapy and breast cancer surgery, menopause status, prior and synchronous malignancy, tumor stage, etc.). The age attribute of ${{\textbf {m}}}_{{cln}}$ is normalized in the range [0,1] using min-max normalization, and other attributes are used as categorical integers. For ${{\textbf {m}}}_{{cnv}}$ modality, we select GISTIC2 thresholded 24,776 genes with estimated values − 2, − 1, 0, 1, 2, representing homozygous deletion, single copy deletion, diploid normal copy, low-level copy number amplification, or high-level copy number amplification, respectively. For ${{\textbf {m}}}_{{dna}}$ modality, we select 23,381 common DNA methylation features from Illumina Infinium HumanMethylation27 and HumanMethylation450 platforms. For ${{\textbf {m}}}_{{mir}}$ modality, we select 1883 common miRBase accession numbers between IlluminaGA and IlluminaHiseq of miRNA mature strand expression RNAseq. For ${{\textbf {m}}}_{{mrna}}$ modality, we select 20,530 genes representing the gene expression values for each patient. The selected genes are further discretized^16^ with values − 1, 0, and 1 representing under-expressed, baseline, and over-expressed genes. We observed certain missing values in the raw data and handled them as follows; if a particular feature had more than 10% missing values, we discarded them, else the weighted nearest neighbor approach^27^ was employed for the estimation of missing values. Finally, the shapes of the raw data are $1236 \times 21$, $1079 \times 24,776$, $1227 \times 23,381$, $1188 \times 1883$, and $1215 \times 20,530$ for ${{\textbf {m}}}_{{cln}}$, ${{\textbf {m}}}_{{cnv}}$, ${{\textbf {m}}}_{{dna}}$, ${{\textbf {m}}}_{{mir}}$, and ${{\textbf {m}}}_{{mrna}}$ modalities, respectively. To handle the curse of dimensionality arising due to high dimensional ${{\textbf {m}}}_{{cnv}}$ and ${{\textbf {m}}}_{{mrna}}$ modalities, or its combinations (bi, tri, quad, pent, hex) with other modalities, we select the top 500 attributes as the best representative subset for the given sample space to acquire the variance higher than 98% throughout the entire cancer patients present in the respective modalities. We discarded the attributes whose values are similar or equal for all patients because these attributes are not helpful for the machine learning classifiers. On the contrary, these attributes can also be bottlenecks for our classification task.

To represent and encode ${{\textbf {m}}}_{{wsi}}$ modality, we needed to develop deep learning methods that can effectively extract hidden informative features from WSIs. However, the high resolution of WSIs makes it difficult to learn from them. Thus, there must be an element of stochastic sampling and filtering involved. In this work, we use a relatively simple approach to sample WSIs. We sample 256 $\times$ 256 pixel patches at the highest resolution using “PyHIST: A Histological Image Segmentation Tool”^28^. Then, we select the top 20% of the generated patches (or 40 patches) with the highest RGB density as ROIs; this ensures that ‘non-representative’ patches belonging to white-space are ignored, and densest tiles include more cells for further investigations^24^. These 40 ROIs represent, on average, 15% of the tissue region within the WSI. Each of these 40 patches is passed through a more profound but less complex CNN, ResNet152^29^ pre-trained over ImageNet dataset^30^ to get the hidden informative features of 2048 dimensions from the last hidden layer.

With the help of survival distribution details extracted from clinical profiles of patients and a 5-year survival cut-off, our problem is defined as the binary classification task as long-term survivors (labeled as 0) and short-term survivors (labeled as 1).

### Methods

The study of multi-modal survival classification task of breast cancer patients is performed through machine learning classifiers such as RBF SVM, (${rbf}\_{{svm}}$) linear SVM (${linear}\_{{svm}}$), polynomial SVM (${polynomial}\_{{svm}}$), sigmoid SVM (${sigmoid}\_{{svm}}$) and Random Forest (*RF*). The low sample size and high-class imbalance of the TCGA-BRCA dataset cause the deep neural networks to lose their generalization abilities resulting in overfitting. Hence, we limit our study to machine learning classifiers only.

**Figure 1:**
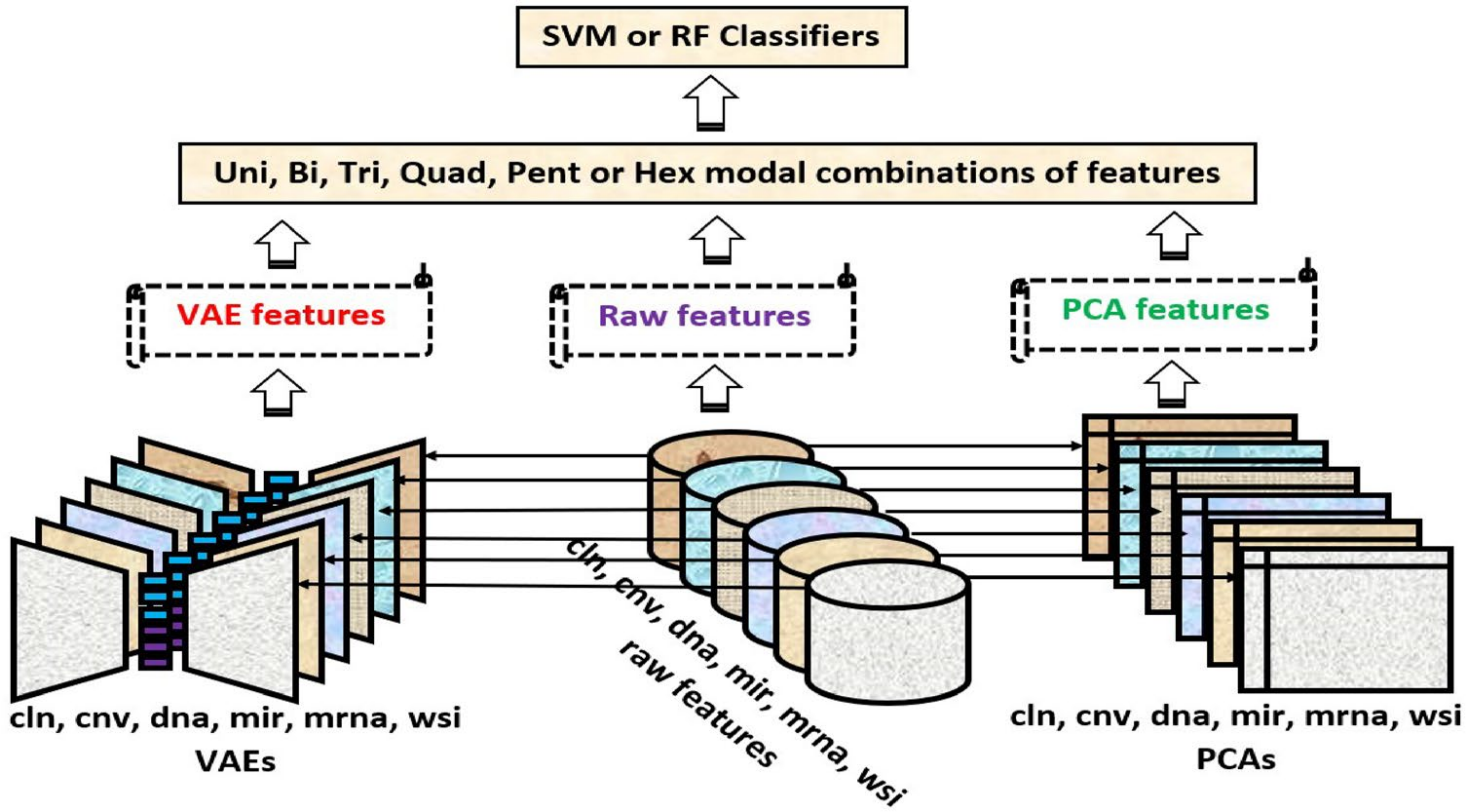
End-to-end flow chart of the proposed breast cancer survival classifiers.

We use three sets of features in the proposed work for our classification. The first set is the selected raw features contributing to 98% variance throughout the instances. It is evident from the dataset section that each uni-modality (except ${{\textbf {m}}}_{{cln}}$) or its multi-modal combinations have high-dimensional feature space and low sample size. The dataset with feature space larger than the number of samples usually suffers from the curse of dimensionality problem^31^ during machine learning training. Acording to the situation, it is important to generate the latent feature with reduced dimensions without losing the relevant information. Apart from raw features, the second feature set is the PCA projected features with reduced dimensions, and the last feature set is extracted from the log-cosh VAE. The final prediction model can be visualized with the flow chart given in Fig. 1.

#### PCA as dimensionality reduction

As PCA is the traditional yet effective dimensionality reduction technique for high dimensional datasets with its ability to project a large number of features to small feature space while preserving as much information as possible, it has been widely used for bioinformatics studies^17,32^. It can predict a sequence of the best linear combinations based on the original attributes of a certain set and reveals a new and reduced set of variables, determined to be the principal components, while also ensuring that little data from the original set is excluded from the analysis. The principal components are ordered according to the highest variance based on the original attributes^33^. In PCA-based feature reduction, we preserve 95% variance among the final selected features for all the uni-modalities. The selected PCA features of ${{\textbf {m}}}_{{cnv}}$ are derived from the linear combination of cnv raw features responsible for the highest variance in the homozygous deletion, single copy deletion, diploid normal copy, low-level copy number amplification, high-level copy number amplification. The selected PCA features of ${{\textbf {m}}}_{{dna}}$ are derived from the linear combination of dna raw features responsible for the highest variance in the ratio of the intensity of the methylated bead type to the combined locus intensity, representing hypermethylation and hypomethylation of the DNA. The selected PCA features of ${{\textbf {m}}}_{{mrna}}$ are derived from the linear combination of mrna raw features responsible for the highest variance in the under-expressed, baseline, and over-expressed genes. Similarly, ${{\textbf {m}}}_{{cln}}$ ${{\textbf {m}}}_{{mir}}$ and ${{\textbf {m}}}_{{wsi}}$ also have the linear combination of their raw features as final PCA features.

For ${{\textbf {m}}}_{{wsi}}$, we projected the hidden features embeddings of pre-trained ResNet152 into the feature space of 512 dimensions with the help of PCA. Further, we concatenated embeddings of all 40 patches to generate the WSI features of size 20,480 for each patient. In the WSI case also, the machine learning model’s training can not happen due to the small number of samples and the large feature space. So, we further reduced the features to 800 dimensions with the help of PCA while preserving 95% variance. Finally, we have the dataset of size 1068 $\times$ 800 for ${{\textbf {m}}}_{{wsi}}$ modality.

#### Log-cosh VAE as dimensionality reduction

**Figure 2:**
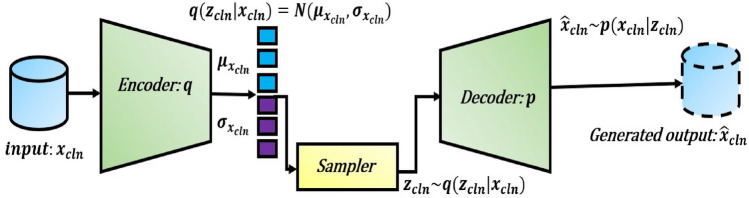
Architecture of the VAE for clinical modality.

In *VAE* based feature reduction technique, we design variational autoencoders (VAEs) with log-cosh loss, $VAE_{cln}$, $VAE_{cnv}$, $VAE_{dna}$, $VAE_{mir}$, $VAE_{mrna}$, and $VAE_{wsi}$ for corresponding modalities ${{\textbf {m}}}_{{cln}}$, ${{\textbf {m}}}_{{cnv}}$, ${{\textbf {m}}}_{{dna}}$, ${{\textbf {m}}}_{{mir}}$, ${{\textbf {m}}}_{{mrna}}$, and ${{\textbf {m}}}_{{wsi}}$, respectively. A simple autoencoder neural network is powerful enough to project any *N*-dimensional data to its *L*, latent dimensions with the highest degree of freedom. But, the high degree of freedom causes the autoencoder to overfit while minimizing the reconstruction loss and generating more specified (less regularized) latent features. To ensure the regularity of the latent space and an enhanced decoder for the regeneration of the raw data, VAE introduces explicit regularization during the training process. Here, we encode the uni-modal raw features as a normal distribution over latent space. In the process of optimizing the regularisation term, Kulback-Leibler divergence between the returned distribution and a standard normal distribution, the encoder is enforced to return a distribution resembling a standard normal distribution by learning the mean and variance of the normal distribution. A random point is sampled from the latent space for reconstruction using a decoder, and reconstruction loss is computed. This can be visualized with the Fig. 2, and the mathematical foundation of the ${VAE}_{cln}$ presented below.

Lets say, the encoder (*q*) of ${VAE}_{cln}$ encodes the raw cln features $x_{cln}$ to latent space $z_{cln}\sim q_{\phi }\left( z_{cln}|x_{cln}\right)$, while the decoder (*p*) reconstructs the raw features as ${\hat{x}}_{cln}\sim p_{\theta }\left( x_{cln}|z_{cln}\right)$ from the latent space $z_{cln}$. Here, $\phi$ and $\theta$ are the encoder and decoder parameters, respectively. The ${VAE}_{cln}$ is designed with two objectives: first is to match the decoded data point ${\hat{x}}_{cln}$ with real clinical features, $x_{cln}$, and second is to maintain the posterior distribution $q_{\phi }\left( z_{cln}|x_{cln}\right)$ to a given prior distribution (standard normal distribution) $p\left( z_{cln}\right)$. So, the VAE is trained with the objective to minimize the following loss function:

1 $$\begin{aligned} \begin{aligned} {\mathscr {L}}\left( \theta ,\phi ,x_{cln}\right) = -{\mathbb {E}}_{q_{\phi }(z_{cln}|x_{cln})} log p_{\theta }(x_{cln}|z_{cln})+D_{KL}(q_{\phi }(z_{cln}|x_{cln})||p(z_{cln})). \end{aligned} \end{aligned}$$

The first term in Eq. (1) represents the reconstruction loss, which becomes $L_{2}$ loss if the decoder predicts the Gaussian distribution $p_{\theta }\left( x_{cln}|z_{cln}\right) \propto \exp (-{\parallel x_{cln}-{\hat{x}}_{cln} \parallel }_{2}^2)$, and $L_{1}$ loss if decoder predicts the zero-mean Laplacian distribution $p_{\theta }\left( x_{cln}|z_{cln}\right) \propto \exp (-{\parallel x_{cln}-{\hat{x}}_{cln} \parallel }_{1})$. The second term tries to match the distribution of latent space to the prior distribution $p(z_{cln})$, which defaults to the standard normal distribution.

If the squared $L_{2}$ loss is incorporated as reconstruction loss, then the Eq. (1) is dominated by the second term (KL divergence term), as $L_{2}$ penalizes small reconstruction errors too lightly, and reconstruction accuracy deteriorates. If the $L_{1}$ loss is incorporated, the larger reconstruction error is penalized too much and harms the optimization of latent space. Another major issue with $L_{1}$ loss is that it is non-differentiable at 0, causing oscillation between $\pm 1$.

To overcome the limitations of $L_{1}$ and $L_{2}$ reconstruction losses, we use log-cosh as the reconstruction loss of the proposed VAE, which takes the benefits of both squared $L_{2}$ loss and $L_{1}$ loss. It also satisfies the basic motivation of VAE to have a balance between reconstruction accuracy and regular latent space generation. The log-cosh loss is defined as follows:

2 $$\begin{aligned} \begin{aligned} {\mathcal {L}}_{logcosh}(x,{\hat{x}})=\sum _{i}f(x_{i}-{\hat{x}}_{i},a)=\frac{1}{a}\sum _{i}log(cosh(a(x_{i}-{\hat{x}}_{i}))),\\ where, cosh(a(x_{i}-{\hat{x}}_{i})) = \frac{\exp [{a(x_{i}-{\hat{x}}_{i})}]+\exp [{-a(x_{i}-{\hat{x}}_{i})}]}{2} \end{aligned} \end{aligned}$$

Here, $a\in \mathcal {R^+}$ is a hyper-parameter. Finally, the proposed VAE is trained on the regularization term (Kulback–Leibler divergence) and reconstruction term (logcosh). $VAE_{cln}$, $VAE_{cnv}$, $VAE_{dna}$, $VAE_{mir}$, $VAE_{mrna}$, and $VAE_{wsi}$ are optimized with motive of minimizing the following equations, respectively:

3 $$\begin{aligned} \begin{aligned} {\mathcal {L}}_{modality} = {\mathcal {L}}_{logcosh}(x_{modality},{\hat{x}}_{modality})+D_{KL}(q_{\phi }(z_{modality}|x_{modality})||p(z_{modality}))\\ where, modality \in \{cln,cnv,dna,mir,mrna,wsi\}. \end{aligned} \end{aligned}$$

The latent feature space for $VAE_{cln}$ is 4 and for all other VAEs, it is fixed to 32. We use encoded latent features from each *VAE* and further concatenate them to form the multi-modal feature set towards the final classification goal.

## Results and analysis

In this section, we perform a comparative analysis of SVM based on some popular kernels and the random forest classifier on raw, PCA projected, and VAE extracted features. The evaluation of the proposed framework is carried out using conventional performance measures like accuracy (*Acc*), precision (*Pre*), sensitivity (*Sn*), and *f_1-score*. To calculate these measures, we use four scenarios: if a patient is survived below 5 years and predicted as a short-term survivor; if a patient is survived more than 5 years and predicted as a long-term survivor; if a patient is survived more than 5 years and predicted as a short-term survivor; if a patient is survived less than 5 years and predicted as a long-term survivor as true positive (tp); true negative (tn); false positive (fp) and false negative (fn), respectively.

### Experimental setup

All the experimental analyses in this study are carried out using the system having ubuntu 18.04.5 LTS along with 11 GB of NVIDIA GeForce RTX 2080 Ti and 32 GB RAM. The coding setup is having keras-gpu 2.2.4 with tensorflow-gpu 1.14.0 in backend and python 3.6 bundled in anaconda environment.

In the task of multi-modal breast cancer survival classification, we performed an ablation study for all possible combinations of modalities ranging from uni-modal to Hexa-modal. The learning is performed with machine learning classifiers for

Uni-modal: [ ${{\textbf {m}}}_{{cln}}$, ${{\textbf {m}}}_{{cnv}}$, ${{\textbf {m}}}_{{dna}}$, ${{\textbf {m}}}_{{mir}}$, ${{\textbf {m}}}_{{mrna}}$, ${{\textbf {m}}}_{{wsi}}$ ]; Bi-modal: [ ${{\textbf {m}}}_{{cln\_cnv}}$, ${{\textbf {m}}}_{{cln\_dna}}$, ${{\textbf {m}}}_{{cln\_mir}}$, ${{\textbf {m}}}_{{cln\_mrna}}$, ${{\textbf {m}}}_{{cln\_wsi}}$, ${{\textbf {m}}}_{{cnv\_dna}}$, ${{\textbf {m}}}_{{cnv\_mir}}$, ${{\textbf {m}}}_{{cnv\_mrna}}$, ${{\textbf {m}}}_{{cnv\_wsi}}$, ${{\textbf {m}}}_{{dna\_mir}}$, ${{\textbf {m}}}_{{dna\_mrna}}$, ${{\textbf {m}}}_{{dna\_wsi}}$, ${{\textbf {m}}}_{{mir\_mrna}}$, ${{\textbf {m}}}_{{mir\_wsi}}$, ${{\textbf {m}}}_{{mrna\_wsi}}$. ]; Tri-modal: [ ${{\textbf {m}}}_{{cln\_cnv\_dna}}$, ${{\textbf {m}}}_{{cln\_cnv\_mir}}$, ${{\textbf {m}}}_{{cln\_cnv\_mrna}}$, ${{\textbf {m}}}_{{cln\_cnv\_wsi}}$, ${{\textbf {m}}}_{{cln\_dna\_mir}}$, ${{\textbf {m}}}_{{cln\_dna\_mrna}}$, ${{\textbf {m}}}_{{cln\_dna\_wsi}}$, ${{\textbf {m}}}_{{cln\_mir\_mrna}}$, ${{\textbf {m}}}_{{cln\_mir\_wsi}}$, ${{\textbf {m}}}_{{cln\_mrna\_wsi}}$, ${{\textbf {m}}}_{{cnv\_dna\_mir}}$, ${{\textbf {m}}}_{{cnv\_dna\_mrna}}$, ${{\textbf {m}}}_{{cnv\_dna\_wsi}}$, ${{\textbf {m}}}_{{cnv\_mir\_mrna}}$, ${{\textbf {m}}}_{{cnv\_mir\_wsi}}$, ${{\textbf {m}}}_{{cnv\_mrna\_wsi}}$, ${{\textbf {m}}}_{{dna\_mir\_mrna}}$, ${{\textbf {m}}}_{{dna\_mir\_wsi}}$, ${{\textbf {m}}}_{{dna\_mrna\_wsi}}$, ${{\textbf {m}}}_{{mir\_mrna\_wsi}}$ ]; Quad-modal: [ ${{\textbf {m}}}_{{cln\_cnv\_dna\_mir}}$, ${{\textbf {m}}}_{{cln\_cnv\_dna\_mrna}}$, ${{\textbf {m}}}_{{cln\_cnv\_dna\_wsi}}$, ${{\textbf {m}}}_{{cln\_cnv\_mir\_mrna}}$, ${{\textbf {m}}}_{{cln\_cnv\_mir\_wsi}}$, ${{\textbf {m}}}_{{cln\_cnv\_mrna\_wsi}}$, ${{\textbf {m}}}_{{cln\_dna\_mir\_mrna}}$, ${{\textbf {m}}}_{{cln\_dna\_mir\_wsi}}$, ${{\textbf {m}}}_{{cln\_dna\_mrna\_wsi}}$, ${{\textbf {m}}}_{{cln\_mir\_mrna\_wsi}}$, ${{\textbf {m}}}_{{cnv\_dna\_mir\_mrna}}$, ${{\textbf {m}}}_{{cnv\_dna\_mir\_wsi}}$, ${{\textbf {m}}}_{{cnv\_dna\_mrna\_wsi}}$, ${{\textbf {m}}}_{{cnv\_mir\_mrna\_wsi}}$, ${{\textbf {m}}}_{{dna\_mir\_mrna\_wsi}}$ ]; Penta-modal: [ ${{\textbf {m}}}_{{cln\_cnv\_dna\_mir\_mrna}}$, ${{\textbf {m}}}_{{cln\_cnv\_dna\_mir\_wsi}}$, ${{\textbf {m}}}_{{cln\_cnv\_dna\_mrna\_wsi}}$, ${{\textbf {m}}}_{{cln\_cnv\_mir\_mrna\_wsi}}$, ${{\textbf {m}}}_{{cln\_dna\_mir\_mrna\_wsi}}$, ${{\textbf {m}}}_{{cnv\_dna\_mir\_mrna\_wsi}}$. ]; and Hexa or Multi-modal: [ ${{\textbf {m}}}_{{cln\_cnv\_dna\_mir\_mrna\_wsi}}$] combinations of modalities. Machine learning classifiers are trained in ten-fold stratified cross-validation set-up. Each fold upsamples the training instances of the minority class to discard the impact of high-class imbalance. In the process of moving from uni-modal to multi-modal machine learning classifiers, the increased feature space of the integrated input data raises the concern related to model overfitting. To avoid this problem, our SVMs are trained as soft-margin SVMs with a small regularization parameter. We also incorporated another hyperparameter gamma which is inversely proportional to the number of input features and controls the influence of the individual data points on the decision boundary. The larger the input feature space implies a smaller value of gamma resulting in a more generic decision boundary influenced by more data points. The following subsections provide a comparative analysis between various combinations of modalities on breast cancer survival prediction.

#### Uni-modal

As we have six different uni-modalities (${m_{cln}}$, ${m_{cnv}}$, ${m_{dna}}$, ${m_{mir}}$, ${m_{mrna}}$ and ${m_{wsi}}$) in this study, we will compare the performance with each other using various performance measures as depicted in Table 2. The *polynomial_svm* is the best classifier among other SVM kernels and random forest for uni-modal data. The average ${Acc, f_{1}-score, and\ Sn}$ of this classifier are 0.706, 0.822, and 0.895, respectively, which are obtained for the VAE-extracted features.

As the study is focused on predicting the survival of the patients and concerning treatment planning based on the predictions, we take ${f_{1}-score}$ as the dominating evaluation measure for comparisons. If we compare the performances of various machine learning classifiers on raw, PCA, and VAE extracted features at each modality level:

- then VAE features of cln modality-based *polynomial_svm* outperforms the raw and PCA extracted features of cln modality based random forest classifiers by 2.7% and 1.0%, respectively.
- then cnv VAE feature based *polynomial_svm* outperforms the cnv raw and PCA features based *polynomial_svm* by 5.8% and 8.1%, respectively.
- then dna VAE feature based *polynomial_svm* outperforms raw feature based RF by 4.6% and PCA features based *polynomial_svm* by 1.5%.
- then mir VAE feature based *polynomial_svm* outperforms raw feature based polynomial_svm by 4.8% and PCA feature based *polynomial_svm* has similar performance.
- then mrna VAE feature based *polynomial_svm* is best in terms of ${f_{1}-score}$ (0.824). It outperforms the raw feature based *polynomial_svm* by 2.0% and gives similar performance with PCA feature based *polynomial_svm*.
- then wsi raw, PCA and VAE feature based *rbf_svms* perform similar with ${f_{1}-score}$ of 0.840.

#### Bi-modal

Here, we perform the comparative study of all fifteen $(^6C_2)$ bi-modal combinations of features generated from the above six uni-modalities. Among all possible bi-modal combinations, *rbf_svm* for mir_wsi combination of raw features; *RF* for dna_wsi combination of PCA features; and *RF* for cnv_mrna combination of VAE features are the best classifiers with ${Acc, f_{1}-score, Sn, and\ Pre}$ values of 0.771, 0.871, 1.00, and 0.771; 0.774, 0.872, 1.00, and 0.774; and 0.776, 0.872, 1.00, and 0.774, respectively. Further, if we compare the average of all bi-modal combinations for raw, PCA, and VAE features, then VAE extracted feature vector-based SVMs, and RF classifiers outperform the other two. The average ${Acc, f_{1}-score, Sn, and\ Pre}$ values of the VAE-based RF classifier are 0.759, 0.862, 0.986, and 0.766; respectively, while PCA extracted feature vector-based RF attains 0.755, 0.858, 0.968, and 0.771 and raw feature-based RF attains 0.738, 0.847, 0.947, and 0.766 values for these metrics, respectively. Similarly, VAE feature-based *rbf_svm* outperforms raw, and PCA feature-based *rbf_svm* by 5.6% and 4.0% and VAE feature-based *polynomial_svm* outperforms raw and PCA feature based *polynomial_svm* by 9.6% and 14.1%, in terms of average $f_{1}-score$. The average results for all bi-modal performance measures are depicted in Table 2.

#### Tri-modal

Here, we perform the comparative study of all twenty $(^6C_3)$ tri-modal combinations generated from the above six uni-modalities. Among all possible tri-modal combinations, *rbf_svm* for cln_cnv_wsi, cln_mir_wsi, cnv_dna_wsi, cnv_mrna_wsi and mir_mrna_wsi combinations of raw features; *RF* for dna_mrna_wsi combination of PCA features; and *polynomial_svm* for cln_mrna_wsi and dna_mrna_wsi combinations of VAE features are best classifiers. The attained ${Acc, f_{1}-score, Sn, and\ Pre}$ values for raw feature-based *rbf_svm* are 0.771, 0.871, 1.00, and 0.771; for PCA feature-based *RF* and VAE feature-based *polynomial_svm* are 0.774, 0.872, 1.00 and 0.774, respectively. Further, we compare the average of all tri-modal combinations for raw, PCA-extracted, and VAE-extracted features. The VAE-extracted feature-based *RF* classifier outperforms the other two. The average ${Acc, f_{1}-score, Sn, and\ Pre}$ values attained by VAE extracted feature-based *RF* classifier are 0.763, 0.865, 0.991, and 0.767, respectively, while raw and PCA extracted feature set-based random *RF* attains values of 0.739, 0.848, 0.949, and 0.766; and 0.755, 0.857, 0.964, 0.772 for these measures, respectively. The dominating measure, ${f_{1}-score}$, for VAE feature-based *rbf_svm* shows 3.6% and 2.2% improvements over raw and PCA feature-based *rbf_svm*, while VAE feature-based *polynomial_svm* outperforms raw and PCA feature based *polynomial_svm* by 7.2% and 8.9%, respectively. The average results for all tri-modal performance measures are depicted in Table 2.

#### Quad-modal

Here, we perform the comparative study of all fifteen $(^6C_4)$ quad-modal combinations generated from the above six uni-modalities. Among all possible quad-modal combinations, *rbf_svm* for cln_cnv_dna_wsi, cln_cnv_mrna_wsi, cln_mir_mrna_wsi, and cnv_dna_mrna_wsi combinations of raw features; for cln_cnv_dna_wsi, cln_mir_mrna_wsi, cln_cnv_mrna_wsi, and cln_cnv_dna_wsi combinations of PCA features; and *RF* for cln_dna_mrna_wsi combination of VAE features are best classifiers. The attained ${Acc, f_{1}-score, Sn, and\ Pre}$ values for raw and PCA feature-based *rbf_svm* are 0.771, 0.871, 1.00, and 0.771; for VAE feature based *RF* are 0.774, 0.872, 1.00 and 0.774, respectively. Further, if we compare the average of all quad-modal combinations for raw, PCA extracted, and VAE extracted features, then the VAE extracted feature-based *RF* classifier outperforms the other two with average ${Acc, f_{1}-score, Sn, and\ Pre}$ values 0.764, 0.866, 0.995, and 0.767, respectively, while raw and PCA extracted feature based *RF* classifier attained 0.738, 0.847, 0.949, and 0.765; and 0.753, 0.856, 0.957, and 0.775 in terms of these performance measures, respectively. The dominating measure, ${f_{1}-score}$, for VAE feature-based *rbf_svm* shows 1.7% and 0.9% improvements over raw and PCA feature-based *rbf_svm*, while VAE feature based *polynomial_svm* outperforms raw and PCA feature based *polynomial_svm* by 5.1% and 4.1%, respectively. The average results for all quad-modal performance measures are depicted in Table 2.

#### Penta-modal

Here, we perform the comparative study of all six $(^6C_5)$ penta-modal combinations generated from the above six uni-modalities. Among all possible penta-modal combinations, *rbf_svm* for cln_cnv_dna_mrna_wsi combination of raw features; for cln_cnv_dna_mrna_wsi combination of PCA features; and *polynomial_svm* for cln_cnv_dna_mrna_wsi combination of VAE features are best classifiers with ${Acc, f_{1}-score, Sn, and\ Pre}$ values of 0.771, 0.871, 1.00, and 0.771, respectively. Further, if we compare the average of all penta-modal combinations for raw, PCA extracted, and VAE extracted feature sets, then the VAE extracted feature-based *RF* classifier outperforms the other two. The average ${Acc, f_{1}-score, Sn, and\ Pre}$ values of the VAE extracted feature-based *RF* classifier are 0.762, 0.864, 0.991, and 0.766, respectively, while raw and PCA extracted feature based *RF* classifier attains ${Acc, f_{1}-score, Sn, and\ Pre}$ values of 0.742, 0.850, 0.954, and 0.767; and 0.752, 0.855, 0.952, 0.777, respectively. The dominating measure, ${f_{1}-score}$, for VAE feature-based *rbf_svm* shows comparable performance with raw and PCA feature-based *rbf_svm*, while VAE feature-based *polynomial_svm* outperforms raw and PCA feature based *polynomial_svm* by 3.4% and 3.9%, respectively. The average results for all penta-modal performance measures are depicted in Table 2.

#### Hexa-modal or multi-modal

Here, we perform the comparative study by combining the above six uni-modalities altogether. The raw and PCA feature-based *rbf_svm* and VAE extracted feature-based *polynomial_svm* are best among various multi-modal machine learning classifiers and achieves ${Acc, f_{1}-score, Sn, and\ Pre}$ values of 0.769, 0.870, 1.00, and 0.769, respectively. If we compare the multi-modal $f_{1}-score$ of VAE feature-based best classifier with uni-modal VAE feature-based best classifiers, then multi-modal *polynomial_svm* leads ${m_{cln}}$ *polynomial_svm* by 9.8%; ${m_{cnv}}$ *polynomial_svm* by 2.8%; ${m_{dna}}$ *polynomial_svm* by 4.5%; ${m_{mir}}$ *polynomial_svm* by 4.2%; ${m_{mrna}}$ *polynomial_svm* by 4.6%; and ${m_{wsi}}$ *polynomial_svm* by 3.0%. The gains of multi-modal over uni-modal measures establish the significance of all six modalities in the breast cancer survival prediction task. The detailed results can be visualized from Table 2.

**Table 2:** Performance measures of machine learning classifiers for breast cancer survival prediction considering various combinations of modalities.

	Classifiers	Rbf_svm			Polynomial_svm			Sigmoid_svm			Random forest		
	Features	Raw	PCA	VAE	Raw	PCA	VAE	Raw	PCA	VAE	Raw	PCA	VAE
Uni	Acc	0.635	0.651	0.643	0.661	0.679	**0.706**	0.513	0.522	0.482	0.656	0.667	0.661
	f1-score	0.749	0.769	0.763	0.778	0.794	**0.822**	0.615	0.628	0.579	0.777	0.786	0.783
	Sn	0.732	0.768	0.777	0.790	0.831	**0.895**	0.512	0.528	0.467	0.788	0.807	0.804
	Pre	0.777	0.774	0.760	0.772	0.769	0.761	0.772	0.776	0.762	0.768	0.767	0.764
Bi	Acc	0.670	0.685	0.725	0.646	0.620	0.747	0.515	0.523	0.464	0.739	0.755	**0.759**
	f1-score	0.780	0.797	0.837	0.757	0.712	0.853	0.620	0.631	0.551	0.847	0.858	**0.862**
	Sn	0.799	0.836	0.924	0.761	0.723	0.966	0.520	0.537	0.430	0.948	0.968	**0.986**
	Pre	0.777	0.772	0.764	0.775	0.775	0.765	0.770	0.767	0.766	0.766	0.771	0.766
Tri	Acc	0.705	0.719	0.742	0.682	0.669	**0.767**	0.511	0.515	0.464	0.740	0.755	0.763
	f1-score	0.813	0.827	0.850	0.795	0.779	**0.868**	0.616	0.622	0.554	0.848	0.858	0.865
	Sn	0.867	0.897	0.954	0.831	0.815	**0.999**	0.516	0.525	0.432	0.950	0.965	0.992
	Pre	0.776	0.774	0.767	0.773	0.769	0.767	0.769	0.769	0.769	0.767	0.773	0.768
Quad	Acc	0.734	0.741	0.750	0.705	0.712	**0.768**	0.501	0.504	0.471	0.738	0.754	0.765
	f1-score	0.838	0.846	0.855	0.818	0.828	**0.869**	0.606	0.608	0.563	0.848	0.857	0.867
	Sn	0.920	0.942	0.966	0.876	0.900	**1.000**	0.502	0.504	0.445	0.950	0.958	0.995
	Pre	0.776	0.771	0.768	0.772	0.766	0.768	0.767	0.769	0.767	0.766	0.776	0.767
Penta	Acc	0.753	0.758	0.755	0.726	0.715	**0.769**	0.490	0.499	0.479	0.743	0.753	0.762
	f1-score	0.856	0.861	0.859	0.836	0.830	**0.870**	0.594	0.602	0.574	0.851	0.856	0.865
	Sn	0.963	0.975	0.971	0.917	0.904	**1.000**	0.487	0.493	0.456	0.955	0.953	0.992
	Pre	0.773	0.770	0.770	0.771	0.767	0.769	0.765	0.773	0.775	0.768	0.778	0.767
Hexa	Acc	0.769	0.769	0.760	0.738	0.718	**0.769**	0.485	0.500	0.481	0.745	0.750	0.767
	f1-score	0.870	0.870	0.862	0.847	0.832	**0.870**	0.589	0.600	0.578	0.854	0.854	0.868
	Sn	1.000	1.000	0.975	0.949	0.911	**1.000**	0.481	0.488	0.463	0.962	0.950	0.988
	Pre	0.769	0.769	0.772	0.765	0.766	0.769	0.760	0.780	0.771	0.768	0.776	0.775

Significant values are in bold.

#### Identifying best feature set (Raw or PCA or VAE) and impact of additional modalities

A thorough empirical and ablation study on six different modalities suggests that VAE-extracted features are the best features for solving breast cancer survival prediction goals. Hence, to study the impact and usefulness of multiple modalities, we finalize VAE extracted features. The RF's average performance measures (${Acc, f_{1}-score, Sn, and\ Pre}$) of hexa-modal (multi-modal) are 0.769, 0.870, 1.00, and 0.769, which are 0.7%, 0.6%, 0.9%, and 0.3% higher than penta-modal; 0.5%, 0.4%, 0.5%, and 0.2% higher than quad-modal; 0.6%, 0.5%, 0.8%, and 0.2% higher than tri-modal; 1.1%, 0.8%, 1.4%, and 0.3% higher than bi-modal; 10.8%, 8.7%, 19.7%, and 0.6% higher than uni-modal average performance measures, respectively. Here, we observe the trend that adding extra modalities is gradually decreasing the performance gap of various classifiers. Further, we also investigate the standard deviation of the performance measures for all combinations of modalities and observe those standard deviations of ${Acc, f_{1}-score, Sn, and\ Pre}$ metrics as 2.94%, 2.55%, 5.45%, and 0.68% for uni-modal; 0.80%, 0.49%, 0.91%, and 0.56% for bi-modal; 0.47%, 0.32%, 0.24%, and 0.38% for tri-modal; 0.29%, 0.21%, 0.00%, and 0.29% for quad-modal; 0.23%, 0.17%, 0.00%, and 0.23% for Penta-modal, respectively. The decline in standard deviation values when we add extra modalities supports the fact that multi-modal classifiers are more stable and robust.

## Discussion

In this study, we present the log-cosh VAEs to get the engineered features as compact representations from various modalities, which include images, genetics, and clinical data. Further, we trained and tested SVMs and Random forests over the various multimodal combinations of modalities for breast cancer prognosis prediction tasks. We also validated the superiority of VAE features over PCA and raw features. In our experiments, we observed the trend that the performance measures of different machine learning classifiers improve when we add more modalities as input to the classifiers. The empirical results from Table 2 suggest that polynomial_svm is the best classifier for uni, tri, quad, pent, and hex modalities, while the random forest is superior for bi-modal combination. The findings of our study establish the fact that all six sources are significant for prognosis, and oncologists must consider all of them while planning the treatment of any breast cancer patient. After this establishment, we also validated the stability and robustness of the classifiers for various multi-modal combinations. The gradual decline in the standard deviations of various performance measures for uni, bi, tri, quad, pent, and hex modal classifiers supports that classifiers with more modalities are highly stable. As the VAE features work as more supportive features for classification goals, they still have limitations in interpretability, as they may not have a clear biological meaning.

## Conclusion

Breast cancer and its heterogeneous information coming from complex sources make it difficult for oncologists to sense the severity of the disease and plan the correct treatment path for the patients. As per our knowledge, it is the first study where we use six different modalities for breast cancer survival estimation. We propose various ways to reduce the complexity of these heterogeneous data. The first approach is the feature selection by preserving the variance of 98% throughout the instances, followed by the PCA as dimensionality reduction and log-cosh VAE as the feature extraction technique. We establish that the VAE extracted features are of very low dimensions (4 for clinical and 32 for other modalities) in contrast with raw and PCA feature space, still achieving better results for the breast cancer survival estimation task. Hence, log-cosh VAEs are reliable feature extraction techniques. We conclude this study with the claim that adding extra modalities has not only improved the performance of machine learning classifiers but also enhanced the stability and robustness over different combinations of modalities. It proves the positive significance of all modalities toward breast cancer survival rate prediction and oncologists should always consider all six modalities in their decision-making. Despite the significant performance of the proposed multimodal architectures in breast cancer prognosis over preprocessed TCGA-BRCA dataset, there is a need to test the models on primary data prospectively to understand their behavior and importance during real-time clinical trials of breast cancer patients.

## Data Availability

The dataset used in this study is available at (https://xenabrowser.net/datapages/) and source code can be accessed at (https://github.com/nikhilaryan92/logcoshVAE_brca_surv).
